# Childhood exposure due to the Chernobyl accident and thyroid cancer risk in contaminated areas of Belarus and Russia

**DOI:** 10.1038/sj.bjc.6690545

**Published:** 1999-07

**Authors:** P Jacob, Y Kenigsberg, I Zvonova, G Goulko, E Buglova, W F Heidenreich, A Golovneva, A A Bratilova, V Drozdovitch, J Kruk, G T Pochtennaja, M Balonov, E P Demidchik, H G Paretzke

**Affiliations:** GSF – Institute of Radiation Protection, Neuherberg, D-85764, Germany; Research and Clinical Institute of Radiation Medicine and Endocrinology, Minsk, 220600, Belarus; Research and Technical Center Pro tection, St Petersburg, 197101, Russia; Republican Scientific and Practical Center of Thyroid Tumors, Minsk, 220600, Belarus; Regional Oncological Clinic, Bryansk, 241032, Russia

**Keywords:** Chernobyl, dose reconstruction, iodine-131, radiation risk, thyroid cancer

## Abstract

The thyroid dose due to ^131^I releases during the Chernobyl accident was reconstructed for children and adolescents in two cities and 2122 settlements in Belarus, and in one city and 607 settlements in the Bryansk district of the Russian Federation. In this area, which covers the two high contamination spots in the two countries following the accident, data on thyroid cancer incidence during the period 1991–1995 were analysed in the light of possible increased thyroid surveillance. Two methods of risk analysis were applied: Poisson regression with results for the single settlements and Monte Carlo (MC) calculations for results in larger areas or sub-populations. Best estimates of both methods agreed well. Poisson regression estimates of 95% confidence intervals (CIs) were considerably smaller than the MC results, which allow for extra-Poisson uncertainties due to reconstructed doses and the background thyroid cancer incidence. The excess absolute risk per unit thyroid dose (EARPD) for the birth cohort 1971–1985 by the MC analysis was 2.1 (95% CI 1.0–4.5) cases per 10^4^ person-year Gy. The point estimate is lower by a factor of two than that observed in a pooled study of thyroid cancer risk after external exposures. The excess relative risk per unit thyroid dose was 23 (95% CI 8.6–82) Gy^−1^. No significant differences between countries or cities and rural areas were found. In the lowest dose group of the settlements with an average thyroid dose of 0.05 Gy the risk was statistically significantly elevated. Dependencies of risks on age-at-exposure and on gender are consistent with findings after external exposures. © 1999 Cancer Research Campaign


British Journal of Cancer (1999) 80(9), 1461–1469
© 1999 Cancer Research Campaign
Article no. bjoc.1999.0545

### Childhood exposure due to the Chernobyl accident and thyroid cancer risk in contaminated areas of Belarus and Russia

**P Jacob[1], Y Kenigsberg[2], I Zvonova[3], G Goulko[1], E Buglova[2], WF Heidenreich[1], A Golovneva[2], AA Bratilova[3], V Drozdovitch[2,*], J Kruk[2], GT Pochtennaja[5], M Balonov[3], EP Demidchik[4] and HG Paretzke[1]**

[1]GSF – Institute of Radiation Protection, D-85764 Neuherberg, Germany; [2]Research and Clinical Institute of Radiation Medicine and Endocrinology, 220600 Minsk, Belarus; [3]Research and Technical Center Protection, 197101 St Petersburg, Russia; [4]Republican Scientific and Practical Center of Thyroid Tumors, 220600 Minsk, Belarus; [5]Regional Oncological Clinic, 241032 Bryansk, Russia

**Summary** The thyroid dose due to $^{131}$I releases during the Chernobyl accident was reconstructed for children and adolescents in two cities and 2122 settlements in Belarus, and in one city and 607 settlements in the Bryansk district of the Russian Federation. In this area, which covers the two high contamination spots in the two countries following the accident, data on thyroid cancer incidence during the period 1991–1995 were analysed in the light of possible increased thyroid surveillance. Two methods of risk analysis were applied: Poisson regression with results for the single settlements and Monte Carlo (MC) calculations for results in larger areas or sub-populations. Best estimates of both methods agreed well. Poisson regression estimates of 95% confidence intervals (CIs) were considerably smaller than the MC results, which allow for extra-Poisson uncertainties due to reconstructed doses and the background thyroid cancer incidence. The excess absolute risk per unit thyroid dose (EARPD) for the birth cohort 1971–1985 by the MC analysis was 2.1 (95% CI 1.0–4.5) cases per $10^4$ person-year Gy. The point estimate is lower by a factor of two than that observed in a pooled study of thyroid cancer risk after external exposures. The excess relative risk per unit thyroid dose was 23 (95% CI 8.6–82) $Gy^{-1}$. No significant differences between countries or cities and rural areas were found. In the lowest dose group of the settlements with an average thyroid dose of 0.05 Gy the risk was statistically significantly elevated. Dependencies of risks on age-at-exposure and on gender are consistent with findings after external exposures.

**Keywords:** Chernobyl; dose reconstruction; iodine-131; radiation risk; thyroid cancer

Current knowledge on thyroid cancer risk due to $^{131}$I during childhood or adolescence is limited. Results of three studies have been published for thyroid doses below 10 Gy (Hamilton et al, 1989; Kerber et al, 1993; Hall et al 1996). Higher doses have a large potential for cell killing and are not considered here. The three studies had low statistical power due to small collective thyroid doses in the cohorts (below $4 \times 10^3$ person Gy), and small numbers both of expected (below six) and observed (below or equal to eight) thyroid cancer cases.

A new source of information on the thyroid cancer risk after $^{131}$I exposure is the reactor accident of Chernobyl on 26 April 1986, more than 70% of the thyroid dose of the population in the contaminated area being due to $^{131}$I (Zvonova and Balonov, 1993). In subsequent years, there was a large increase of the thyroid cancer incidence in Belarus (Kazakov et al, 1992; Buglova et al, 1997) and in Ukraine (Likhtarev et al, 1995; Sobolev et al, 1997).

A case-control study with 107 thyroid cancer cases and two matched control groups of similar size indicated a strong relationship between thyroid cancer and radiation dose from the Chernobyl accident (Astakhova et al, 1998). Cohort studies of individuals whose thyroid $^{131}$I activity was measured during the first 2 months after the accident would be expected to give reliable results, although possible drawbacks include:

1. long observation times necessary to gather enough cancer cases to achieve statistical power
2. large uncertainties of the individual thyroid doses due to the unknown thyroid mass at the time of the accident.

These drawbacks can be minimized in aggregate studies: hundreds of excess thyroid cancer cases have already now occurred and average thyroid doses can be reconstructed with a higher reliability than individual thyroid doses. On the other hand, the appropriateness of aggregate studies to derive quantitative information on risk is limited to special cases (e.g. Greenland and Robin, 1994; Sheppard et al, 1996). A linear dose–response relationship and a control of confounding factors are essential for deriving reliable results.

Two aggregate studies (Buglova et al, 1996; Jacob et al, 1998) of the thyroid cancer incidence in the contaminated areas demonstrated the large potential of such analyses to derive quantitative results on the cancer risk after $^{131}$I exposures.

The purpose of this paper is to continue these analyses by:

1. summarizing observations related to regional and temporal variability of thyroid surveillance, which is potentially a large confounding factor for thyroid cancer after Chernobyl, and estimating an uncertainty range for the background incidence
2. discussing the methodology for assessing best estimates and confidence intervals for the risk per unit dose

*Received 17 September 1998*
*Revised 30 November 1998*
*Accepted 28 January 1999*

*Correspondence to:* P Jacob

*Present address: Institute of Power Engineering Problems, 220109 Minsk, Belarus.

3. deriving information on the dependence of the risk on age at exposure and on gender.

### MATERIALS AND METHODS

### Study area and population structure in 1986

The study area covers the two main contamination spots in Belarus and Russia that occurred after the Chernobyl accident (Figure 1). It consists of three cities (Bryansk, Gomel and Minsk), and all settlements in six areas in Bryansk district (Russian Federation), nine areas in Gomel district and eight areas in Mogilev district (both Belarus), including the population of evacuated settlements. The area was chosen because a large amount of $^{131}I$ measurements in human thyroids was available in the three cities and in the six areas of Bryansk district. Further, published data on $^{131}I$ contaminations of the environment and $^{137}Cs$ contaminations of each settlement in the other 17 areas were available as an input to a radioecological model (Drozdovitch et al, 1997).

To estimate the population structure in the study area in Belarus at the time of the accident, the following officially published data were used:

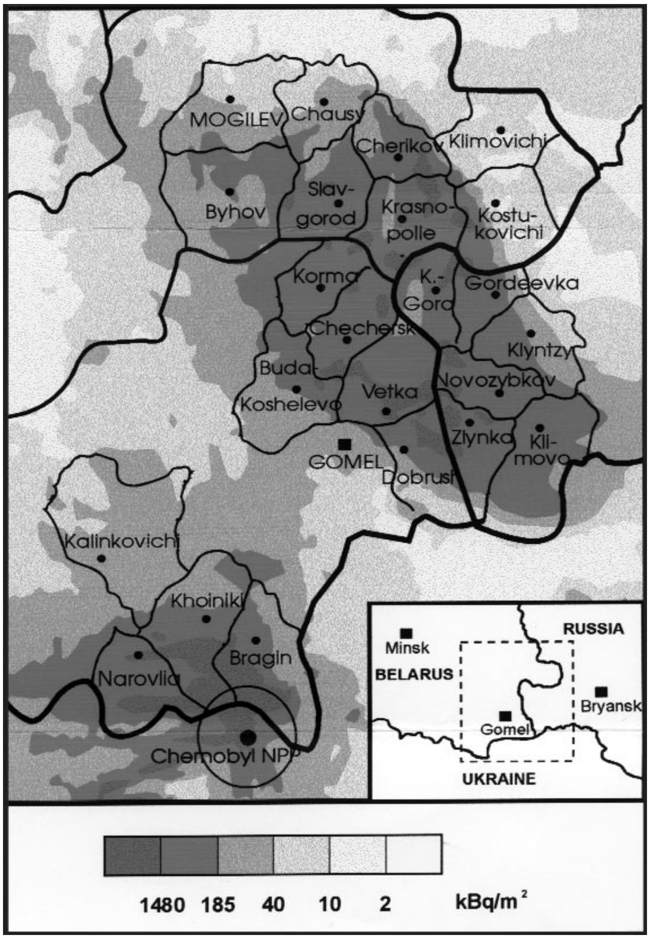

**Figure 1** The study area consisting in Belarus of Minsk city, Gomel city and 2122 settlements in nine areas of Gomel district and in eight areas of Mogilev district, and in Russia of Bryansk city and 607 settlements in six areas of Bryansk district

- total population *n* in 1986 of the cities and towns, and of the villages that were evacuated during May/June 1986
- results of estimation for the population $N_{a,s}$ for age-groups 0, 1–4, 5–9, 10–14, 15–19, ..., and gender *s* in each district in 1986 (performed by the Ministry of Statistic and Analysis)
- total population $n^{89}$ in each settlement according to the census data for 1989
- age- and gender-specific death rates $q^{year}_{i,s}$ for the years 1986, 1987 and 1988.

For cities and towns, the number of people $n_{a,s}$ in 5-year intervals and for gender *s* was estimated according to:

$$n_{a,s} = n \times N_{a,s}/\Sigma_s \Sigma_{a\geq 0} N_{a,s} \qquad (1)$$

In some towns the number of children in 1986 was known. For the calculation of the age and gender structure among children, the summation in (1) extended in these cases only over the first four age intervals. Linear interpolation was used to estimate the population $n_{i,s}$ in 1-year intervals.

Since, except for the evacuated villages, there are no official data about village population in 1986, we have used 1989 census data. Equation (1) and linear interpolation were applied to obtain the population $n^{89}_{i,s}$ in 1-year intervals. Age- and gender-specific survival rates $p^{year}_{i,s}$ were used to calculate the population structure $n^{86}_{i,s}$ according to:

$$n^{86}_{i,s} = n^{89}_{i+3,s}/(p^{88}_{i+2,s} \times p^{87}_{i+1,s} \times p^{86}_{i,s}). \qquad (2)$$

The age and sex structure of the population of the Bryansk district was derived from the census data for 1989, which show the numbers of males and females by 5-year age groups in Bryansk city and in every settlement. These data were used for 1986, with linear interpolation to obtain results for each year of age.

### Thyroid doses in Belarus

The doses reconstructed in the present paper are based on environmental measurements and on a radioecological model (Drozdovitch et al, 1997).

The most important pathway for thyroid exposure to $^{131}I$ was ingestion with contaminated milk. During the time of deposition, leafy vegetables were not well-developed, even in the southern part of Belarus, and iodine ingestion with leafy vegetables was estimated to be in range of several per cents. Thyroid exposure due to inhalation was only important (a) for evacuees shortly after the accident and (b) for persons with radically reduced milk consumption (Zvonova and Balonov, 1993). Therefore, thyroid exposure only due to incorporation of $^{131}I$ with milk is considered here.

Relatively large data sets on measurement results of $^{131}I$ activities in soil, grass and milk samples were available. The following radioecological local parameters were assessed: (a) ratio between activities of $^{131}I$ and $^{137}Cs$ in ground deposition; (b) initial interception of $^{131}I$ by vegetation; (c) elimination rate of $^{131}I$ from grass and milk; and (d) transfer factor for $^{131}I$ from grass to cows' milk. Age-dependent milk consumption rates estimated in a public survey were used in the calculations. Additionally, the influence of applied countermeasures (evacuation; introduction of maximum permissible level of $^{131}I$ activity in milk) was taken into account.

In 91 settlements there were more than 15 measurements per settlement of $^{131}I$ activities in thyroids available for the age group 13–17 (Gavrilin et al, 1992). For 95% of these settlements, the results of the radioecological model and the measurements agree within a factor of 4 (Drozdovitch et al, 1997).

 *© 1999 Cancer Research Campaign*

**Table 1** Average thyroid dose in different age cohorts relative to the average thyroid dose of adults; for Bryansk, the 95% range of the measurement results and the number of measurements are also indicated

| Birth cohort | Belarus (model) | Russia (measurements) |
|---|---|---|
| 1986 | 4.6 | 9.8 (1.6–36)/344 |
| 1980–1985 | 4.1 | 3.6 (1.8–6.9)/2679 |
| 1976–1979 | 2.2 | 1.6 (0.9–2.7)/1365 |
| 1971–1975 | 1.5 | 1.5 (0.9–2.4)/1260 |
| 1968–1970 | 1.1 | 1.2 (0.6–2.4)/474 |
| 1971–1985 | 2.6 | 2.4 (1.4–3.7)/5304 |
| Before 1968 | 1.0 | 1.0 (–)/6615 |

**Table 2** Results of a Poisson regression analysis of the thyroid cancer incidence rate in the period 1991–1995 among the birth cohort 1971–1985 in three cities and 2729 settlements in Belarus and Bryansk district

| Model | Constant term per $10^6$ person-years | Linear term per $10^4$ person-year Gy | Quadratic term per $10^4$ person-year $Gy^2$ |
|---|---|---|---|
| Linear | 11 (4–19) | 1.9 (1.6–2.3) | – |
| Linear–quadratic | 9 (2–17) | 2.2 (1.6–2.7) | –0.2 (–0.4–0.2) |

Values in parentheses indicate 95% confidence intervals.

### Thyroid doses in Bryansk district

Dose reconstruction in Bryansk district was based on measurements of the $^{131}$I activity in thyroids performed in the period 13 May to 13 June 1986. Two types of devices were used for these measurements: the radiodiagnostic device GAMMA and the radiometer SRP-68-01. Both were NaI(T1) scintillation detectors. The diagnostic equipment had a crystal (40 × 40 mm) with a lead collimator, and was operated with a low energy threshold between 200 and 300 keV. The device SRP-68-01 had a crystal (30 × 20 mm) without collimator and with an energy threshold of 25 keV. The number of measurements was 1017 with the radiodiagnostic equipment, and 11 720 with SRP-68-01.

The diagnostic equipment was calibrated using a neck-phantom. Experimentally obtained calibration factors were in a good agreement with values calculated by the Monte Carlo (MC) method (Ulanovsky et al, 1997). Calibration factors for measurements of $^{131}$I in human thyroid with SRP-68-01 were determined by investigations of patients who received $^{131}$I for diagnostic examination. Calibration factors for caesium were determined in investigations with volunteers who incorporated radiocaesium (Kaidanovski and Dolgirev, 1997).

All measurements with GAMMA and some with SRP-68-01 were carried out in two detector positions: the first over the front surface of the neck and the second over the thigh. The second measurement was used for the estimation of the count rate due to radiocaesium distributed in a human body (Zvonova et al, 1998). Individual correction factors were calculated for all double measurements. Average values for each day of examination and for three age groups, 0–7, 8–17 and above 17 years, were determined and applied to measurements for which no second measurement over the thigh was performed.

Taking into account the contribution of extrathyroidal activity reduces thyroid dose estimates by 20–50% for measurements performed with SRP-68–01 in the middle of May and by a factor of 2–3 in the first 10 days of June 1986.

Based on about 1000 measurements of total β activity in milk, the $^{131}$I intake was assumed to be constant within the first 10–15 days, depending on local conditions of cows' grazing. For later times, the $^{131}$I intake was assumed to decrease as the concentration in milk with a half-life of 5 days.

Information on the beginning of the grazing period, and whether and when the $^{131}$I intake was reduced drastically due to taking stable iodine or due to the prohibition of local milk consumption, was obtained by interviewing people and local authorities.

Individual thyroid doses were assessed for 12 732 inhabitants of Bryansk district; 11 300 of these lived in settlements with more than five measurements of the $^{131}$I activity in the thyroid of adults (18 years or older at the time of accident). Individual thyroid doses of children younger then 18 were normalized to arithmetic means of the doses of adults in each settlement. The resulting age dependence was applied to all settlements.

Doses in settlements with fewer than six measurements of $^{131}$I activities in thyroids of adults were obtained by an interpolation procedure based on a correlation of thyroid doses with $^{137}$Cs activities per unit area and with the gamma dose rate in air in the period when the contribution by $^{131}$I was maximal (Zvonova and Balonov, 1993).

### Collection of records about thyroid cancer cases

A database about thyroid cancer cases in Belarus was established at the Research and Clinical Institute of Radiation Medicine and Endocrinology. The following information sources were used:

- medical records for patients with thyroid cancer operated in the Republican Scientific and Practical Center of Thyroid Tumors and in the clinic of the Research and Clinical Institute of Radiation Medicine and Endocrinology,
- the Belorussian Cancer Registry, and
- data from the regional dispensaries of oncology.

**Table 3** Basic data for a risk analysis for thyroid cancer in the period 1991–1995 among the birth cohort 1971–1985 in the study area. Assumed 95% confidence intervals are given in parentheses.

| Area | Children (in thousand) | Average thyroid dose (Gy) | Observed cases | Background cases[a] |
|---|---|---|---|---|
| Bryansk, city | 100 (80–120) | 0.049 (0.025–0.098) | 7 | 4.7 (1.6–7.8) |
| Bryansk, rural | 57 (46–68) | 0.20 (0.10–0.40) | 21 | 2.6 (0.9–4.3) |
| Gomel, city | 111 (89–133) | 0.37 (0.19–0.74) | 69 | 5.2 (1.8–8.6) |
| Gomel, rural | 85 (68–102) | 1.07 (0.54–2.1) | 93 | 3.9 (1.3–6.5) |
| Mogilev, rural | 56 (45–67) | 0.59 (0.30–1.2) | 14 | 2.6 (0.9–4.3) |
| Minsk, city | 342 (274–410) | 0.07 (0.04–0.14) | 39 | 16 (5.4–27) |

[a]Best estimate is assumed to be three times the incidence that was observed in Belarus in the period 1983–1987 for the same age group.

**Table 4** Excess absolute risk per unit thyroid dose in the observation period 1991–1995 among the birth cohort 1971–1985, median values and 95% confidence intervals

| Area | Excess absolute risk per unit dose (per $10^4$ person-year Gy) |
|---|---|
| Bryansk, city | 1.0 (–1.8–4.9) |
| Bryansk, rural | 3.5 (1.3–8.2) |
| Gomel, city | 3.2 (1.5–6.9) |
| Gomel, rural | 2.1 (1.0–4.4) |
| Mogilev, rural | 0.7 (0.2–1.8) |
| Minsk, city | 1.9 (0.5–4.9) |
| Study area in Belarus | 2.1 (1.0–4.4) |
| Study area in Bryansk | 2.7 (0.9–6.5) |
| Cities | 2.6 (1.2–5.8) |
| Rural areas | 1.9 (0.9–3.9) |
| All | 2.1 (1.0–4.5) |

**Table 5** Observed number of thyroid cancer and excess absolute risk per unit thyroid dose (per $10^4$ person-year Gy) in the period 1991–1995 in three cities and 2729 settlements in Belarus and Russia

| Birth period | Boys | Girls | Both |
|---|---|---|---|
| 1968–1970 | 3/0.02 (–1.3–1.4) | 21/2.5 (–1.0–8.4) | 24/1.2 (–0.8–4.4) |
| 1971–1975 | 6/0.2 (–0.4–1.2) | 26/1.4 (–0.3–4.3) | 32/0.8 (–0.2–2.5) |
| 1976–1979 | 18/1.6 (0.6–3.7) | 36/3.0 (1.2–6.9) | 54/2.3 (1.0–5.0) |
| 1980–1985 | 65/2.0 (1.0–4.3) | 92/2.9 (1.4–6.0) | 157/2.5 (1.2–5.0) |
| 1986[a] | 1/0.3 (–0.5–1.4) | 5/1.8 (0.2–5.1) | 6/1.1 (0.2–2.9) |

[a]Born before 31 May 1986. Median values and 95% confidence intervals of 10 000 trials in a Monte Carlo calculation for each category are given.

The Belorussian Cancer Registry collects data from 12 oncological dispensaries of the Republic where primary medical records of cancer patients are being stored. Most of the cases operated in the Republican Scientific and Practical Center of Thyroid Tumors were verified by an international panel of pathologists.

The different information sources were compared to clarify the records for each thyroid cancer case. The most important and labourious task was to identify the address at the time of Chernobyl exposure, because many people under investigation moved after the Chernobyl accident.

People with thyroid cancer incidence from our study area in Russia were operated upon in hospitals of the Bryansk district, or in Moscow or Obninsk. Histological samples are analysed in the clinic where the operation was performed. Subsequently, most of them were checked in specialized clinics in Moscow and Obninsk. Information on thyroid cancer cases among inhabitants of the Bryansk district is collected in the regional oncological register, which was used for the risk analysis.

The record for each thyroid cancer case includes:

- date of birth
- gender
- place of residence at the time of accident
- date of operation.

### Thyroid surveillance and background incidence rate of cancer

In Belarus and Russia, programmes of annual medical examinations were set up shortly after the accident to survey children for thyroid disease. Examinations include palpation of the neck, ultrasound imaging and thyroid hormone testing. In Belarus, 62% of the thyroid cancer cases among children were found in the period 1986–1994 by this programme (Stsjazhko et al, 1995). A main effect of this intense thyroid surveillance of children might be an early diagnosis, contributing to the considerably higher thyroid cancer incidence in Belarus for ages 7–15, if compared to ages 16–22. This is consistent with the relatively high amount (25%) of tumours of size smaller than 1 cm.

Due to an order of the Ministry of Public Health of the former Soviet Union from Autumn 1986, in addition to the thyroid surveillance for children, in areas with $^{137}Cs$ contaminations larger than 555 kBq m$^{-2}$ the adult population is requested to attend an annual clinical examination that includes palpation and ultrasound imaging of the thyroid.

The effect of enhanced thyroid surveillance is expressed in this study by an age-independent factor S on the incidence rate in Belarus in the period 1983–1987, which has been approximated for all persons younger than 27 years by $0.012*(\text{age})^{2,02}$ cases per $10^6$ person-years and similar functions for males and females (Heidenreich et al, 1999).

The following data on thyroid cancer incidence in Belarus after the Chernobyl accident were used to derive an estimate of S:

i. Among those born in Belarus in the period 1987–1997 (not exposed by the Chernobyl accident but being subject to the same thyroid surveillance as those exposed) nine thyroid cancer cases were registered up to the end of 1997. This corresponds to a factor S of 4, which indicates the upper range because of the more intense surveillance for school-children.
ii. A large amount (45%) of the tumours operated in the Republican Scientific and Practical Center of Thyroid Tumours were in a late stage. At least these cases would have become obvious independent of any thyroid surveillance indicating a factor of S not more than 2.

**Table 6** Basic data and excess absolute risk per unit thyroid dose (result of Monte Carlo calculations) for the cancer incidence in the period 1991–1995 among the birth cohort 1971–1985 in three cities and 2729 settlements in Belarus and Russia

| Range of thyroid doses (Gy) | Average thyroid dose (Gy) | Person-years (in thousands) | Observed cases | Background cases[a] | Excess absolute risk per unit dose (per $10^4$ person-year Gy) |
|---|---|---|---|---|---|
| ≤ 0.1 | 0.05 | 1756 | 38 | 16 | 2.6 (0.5–6.7) |
| 0.1–0.5 | 0.21 | 1398 | 65 | 13 | 1.9 (0.8–4.1) |
| 0.5–1.0 | 0.68 | 386 | 52 | 3.6 | 2.0 (0.9–4.2) |
| 1.0–2.0 | 1.4 | 158 | 50 | 1.5 | 2.3 (1.1–4.9) |
| > 2.0 | 3.0 | 56 | 38 | 0.5 | 2.4 (1.1–5.1) |

[a]Best estimate is three times the incidence that was observed in Belarus in the period 1983–1987 for the same age group.

 © 1999 Cancer Research Campaign iii. Among the birth cohort 1968–1986 in Belarus, 23 thyroid cancer cases were observed in the period 1986–1988, which is before the onset of the Chernobyl induced cases. This corresponds to a factor S of 1.5, which indicates the lower range because in the early period after the accident the equipment for thyroid surveillance was not as good as in the period of interest (1991–1995) for this study.

A value of 3 was assumed as the best estimate of the factor S for the study area. For the birth cohort 1971–1985 and the observation period 1991–1995 this corresponds to a background risk of about nine cases per $10^6$ person-years. The background risk to males is about a factor of three smaller than for females.

### Risk analysis

For each of the 2732 cities and settlements in the study area, the average age-specific thyroid dose $D_i$, the person-years at risk $PY_i$, and the number $L_i$ of cancer cases in the period 1991–1995 were the input data for the risk analysis. The following model is used for the number of cases $\Lambda_i$ in each settlement:

$$\Lambda_i = PY_i * (r_0 + r_1 *D_i + r_2 *D_i^2),$$

where $r_0$ is the background risk, $r_1$ the EARPD, and $r_2$ tests for non-linear dose-dependencies. The linear model has $r_2 = 0$. The excess relative risk per unit thyroid dose (ERRPD) is defined by $r_1/r_0$.

These modelled numbers $\Lambda_i$ are compared with the observed number of thyroid cancer cases $L_i$ in each settlement by Poisson regression (McCullagh and Nelder, 1991) minimizing the deviance

$$d = -2\Sigma^k_{i=1} (L_i - \Lambda_i + L_i \, 1n \, \Lambda_i/L_i).$$

The 95% confidence bounds of the estimated parameters are calculated by the profile likelihood method: If $d_{min}$ is the minimum of the deviance, then for each of the parameters the range is calculated in which the deviance is smaller than $d_{min}$+4.00, if the other parameters are still allowed to vary in order to minimize the deviance. The calculations were performed with the program MINUIT (James, 1994).

Since the standard Poisson regression does not take into account uncertainties of population data and dose estimates, an alternative procedure was adopted to derive risk factors and their confidence intervals. The excess risk per unit thyroid dose was calculated for groups of the settlements by MC calculations with the program Crystal Ball (Decisioneering, Denver, CO, USA). Based on the material discussed in the previous sections, the following uncertainty distributions of the parameters were used:

i. person-years at risk were assumed to be normally distributed with a standard deviation of 10%
ii. estimates of average thyroid doses for larger population groups or areas were assumed to be lognormally distributed with a geometrical standard deviation of sqrt(2)
iii. the number L of observed cancer cases in the sub-cohorts of interest was assumed to be a sample of a variable with a Poisson distribution
iv. the factor S for the background incidence rate was assumed to be normally distributed with a standard deviation of 33%.

No correlations between the four input parameters were taken into account. For each calculation, 10 000 runs were performed, median values and 2.5 and 97.5 percentiles were calculated. Sensitivity analyses were performed to determine the contributions of the four input variables to the uncertainty of the excess risks per unit dose.

Excess risks were calculated for the birth cohort 1971–1985 (age 0.4–15.4 years at the time of exposure) for different sub-areas and dose groups. For the whole study area calculations were performed for both genders and for birth cohorts 1986 (from 1 January to 31 May), 1980–1985 (children at the time of exposure and at the time of operation), 1976–1979 (intermediate group), 1971–1975 (children at time of exposure and adolescents or young adults at time of operation) and 1968–1970 (adolescents at time of exposure and young adults at time of operation).

### RESULTS

### Dose distributions

Average thyroid doses of small children are about a factor of 5 higher than average doses of adults. The results of the radio-ecological model agree well with the measured 95% ranges of the individual relative doses (Table 1).

In the Belarus study area, 95% of the birth cohort 1971–1985 (children at the time of the accident) belong to settlements with age-specific thyroid doses in the range of 0.035–2 Gy, in Bryansk in the range 0.02–0.5 Gy (Figure 2). The dose distribution of the population is at lower thyroid doses than the distributions of the cancer cases and of the collective dose. The similarity of the latter two distributions indicates the high correlation of thyroid dose and cancer cases. For doses below 1 Gy, the distribution of the cases is shifted to lower doses if compared to the distribution of the collective dose, indicating the contribution of thyroid cancer cases that occur independently of the dose due to the Chernobyl accident.

### Poisson regression versus Monte Carlo calculations

Poisson regression analysis of the thyroid cancer incidence in the period 1991–1995 among the birth cohort 1971–1985 in three cities and 2729 settlements in Belarus and Bryansk district with linear and linear–quadratic models gave best estimates for the thyroid cancer incidence rate among unexposed person of 11 and nine cases per $10^6$ person-years respectively (Table 2), in full accordance with the assumption of a value of 3 for the screening factor 5. The large confidence intervals indicate that the study cohort is not suited for a quantitative assessment of the background incidence rate.

The linear term is about two excess cases per $10^4$ person-year Gy. It is lower in the linear model than in the linear–quadratic model, although there is no statistically significant difference.

The best estimate for the quadratic term is small (–0.2 cases per $10^4$ person-year $Gy^2$). The difference from zero is not statistically significant.

The MC calculation resulted in an excess absolute risk per unit thyroid dose (EARPD) of 2.1 (95% CI 1.0–4.5) cases per $10^4$ person-year Gy, agreeing well with the best estimates according to the Poisson regression. As expected, the CI according to the MC calculation is considerably larger than for the Poisson regression. If in the MC calculation only the Poisson error of the observed number of cases is taken into account and all other uncertainties are neglected, the calculation results in a similar CI as the Poisson regression. In the following, only the MC method was applied to calculate excess risks and their CIs.

© 1999 Cancer Research Campaign

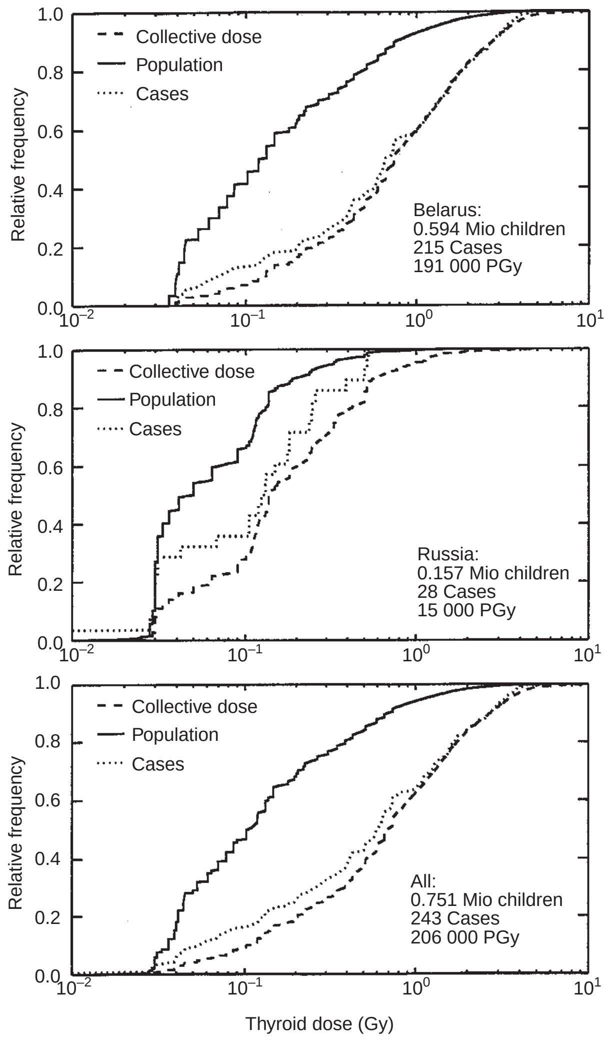

**Figure 2** Dose distributions among the birth cohort 1971–1985 in the study area

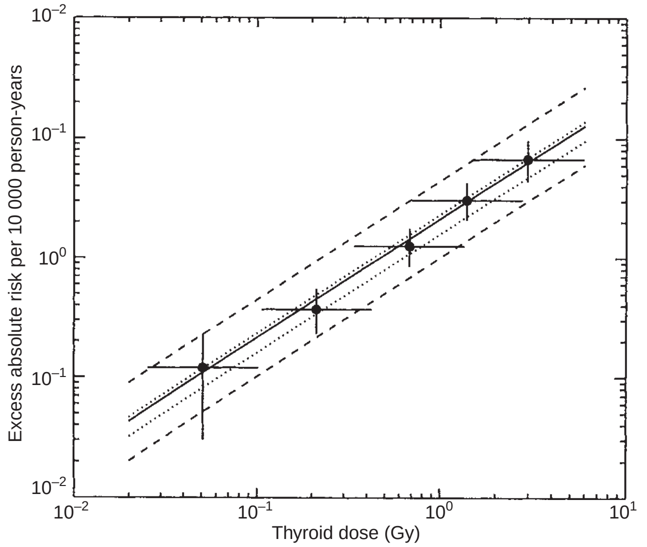

**Figure 3** Excess thyroid cancer incidence rate in the period 1991–1995 among the birth cohort 1971–1985 in three cities and 2729 settlements in Belarus and Russia: Observation for inhabitants of cities and settlements with average thyroid doses of ≤ 0.1, 0.1–0.5, 0.5–1.0, 1–2 and > 2 Gy (points and 95% confidence error bars), linear models according to Poisson regression (95% confidence dotted lines) and Monte Carlo simulation (solid line and 95% confidence broken lines)

### Excess thyroid cancer risk per unit thyroid dose

Basic data for the birth cohort 1971–1985 in six sub-areas of the study area are shown in Table 3. The best estimates for the EARPD in ten combinations of the sub-areas were in the range 0.7–3.5 cases per $10^4$ person-year Gy. The 95% CIs of the EARPD for all sub-areas overlap (Table 4). There is no statistically significant difference either between Belarus and Bryansk district, or between cities and rural areas. In eight of the 11 categories analysed, the uncertainty of the dose assessment dominated with a contribution of 54–86% the variance of EARPD; in the rural areas of Mogilev district and in Minsk city it contributed 45% and 41% respectively. The Poisson distribution of the observed cases was the dominating contributor (67%) for Bryansk city, for rural areas in Mogilev district it contributed (46%), and in the other cases less than 34%. The uncertainty of the background incidence rate contributed at most 22% (Bryansk city), and the uncertainty of the person-years at risk was always negligible.

The excess relative risk per unit thyroid dose (ERRPD) for the birth cohort 1971–1985 in the study area was 23 (95% CI 8.6–82) $Gy^{-1}$. The largest contributors to the uncertainty were the uncertainties of the background incidence rate (53%) and of the thyroid doses (40%).

In the birth cohort 1976–1985 the best estimate of the EARPD for males is by a factor of 1.7 lower than that for females (Table 5). The EARPD for females in the birth cohorts 1971–1975 and 1968–1970 is lower than for the birth cohort 1976–1985, by a factor of 2 and 1.2 respectively. The differences are not statistically significant. For boys in the birth cohort 1968–1970, the number of observed thyroid cancer cases is comparable to the estimated background. Taking both genders together, the EARPD of those born in the period 1976–1985 is by a factor of 2 higher than of those born in the first 5 months of 1986 and in the period 1968–1975.

For the birth cohort 1971–1985, the ERRPD of boys was 39 $Gy^{-1}$ and by a factor of 2 higher than the ERRPD for girls. The ERRPD is highest for young children at time of exposure. For the birth cohort 1976–1979 it is lower by a factor of 2, and for the birth cohort 1968–1975 it is lower by a factor of 10.

The best estimates for the excess absolute risk in five dose groups lie within the narrow uncertainty band of the linear risk model obtained by the Poisson regression (Figure 3). Accordingly, the EARPD in all five dose groups is very similar (Table 6). The lowest dose group has an average thyroid dose of 0.05 Gy and the EARPD is significantly different from zero.

### DISCUSSION

### Diagnostic $^{131}I$ administration

Among these studies on thyroid cancer after diagnostic administration of $^{131}I$ the Swedish study (Hall et al, 1996) had the largest collective thyroid dose. Hall et al concluded: ‘Among those under age 20 years when $^{131}I$ was administered, a small excess risk …

© 1999 Cancer Research Campaign

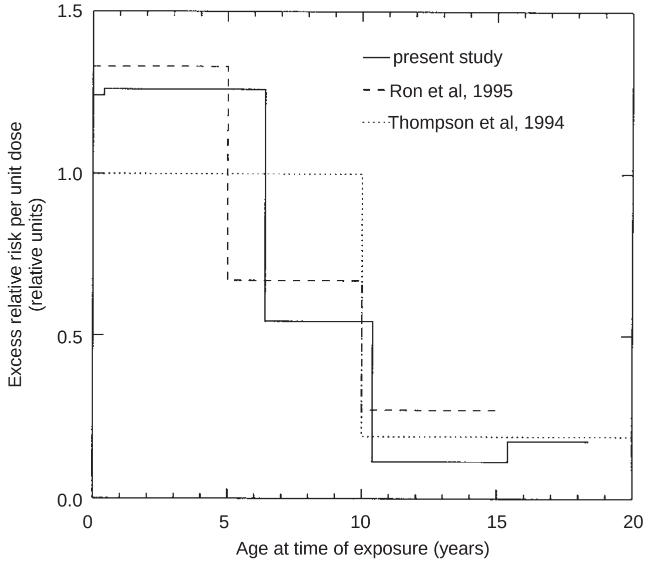

**Figure 4** ERRPD for different age-at-exposure groups, normalized on the age group 0–10 years was about 2–10 times lower than that predicted from data for the A-bomb survivors'. There are two reasons the small risk found in this study should not be generalized without caution:

1. Only 300 of the 2408 in the cohort under age 20 were younger than 10 years (Hall, personal communication). Among the A-bomb survivors the thyroid cancer incidence among those exposed before the age of 10 was increased by a factor of three more than the increase in incidence in the age group 10–19 (Thompson et al, 1994).
2. Estimates of thyroid doses after $^{131}$I administration have a large uncertainty if the mass of the individual thyroid is not known. Hall et al (1996) had no information on the individual thyroid mass for 52% of the cohort. Large uncertainties in dose estimates are known to result in an underestimation of risk coefficients, if they are not corrected for (Pierce et al, 1990; Thomas et al, 1993).

Uncertainties in thyroid dose estimates may also explain the absence of a dose dependency in the study of Hall et al (1996). In contrast to the whole study cohort, for the sub-cohort with known thyroid masses a dose dependency was seen: the 95% confidence of the standardized incidence ratio (SIR) in the lowest dose group excluded the SIR in the highest dose group and vice versa.

Finally, the range of the factor 2–10 in the above statement was based on Poisson statistics. Accounting for other uncertainties like those in the dose estimates would enlarge the confidence interval.

In summary, the non-conclusive findings of studies of thyroid cancer after $^{131}$I administration do not question the results of the present study.

### Thyroid surveillance and background incidence rate of cancer

The assumed screening factor of 3 may be compared with other findings reported in the literature. It was argued (Beral and Reeves, 1992) that at autopsy, the prevalence of occult papillary carcinoma is 2% at age 0–15 and 22% at age 16–30 (Franssila and Harach, 1986), suggesting that many of the thyroid cancer cases observed after Chernobyl might be occult. However, pathological examinations since then have shown that the thyroid cancer cases reported after Chernobyl are of aggressive nature in contrast to occult carcinoma (Williams, 1994; Pacini, 1997).

The magnitude of screening effects on thyroid cancer incidence depends strongly on the conditions of the study:

1. A recall and screening programme of the Michael Reese Hospital (MRH), Chicago, of patients who were treated with head and neck radiation in the period 1939–1962 enhanced the thyroid cancer incidence in the period of most intensive screening (1974–1979) by a factor of 7 for the whole cohort and by a factor of 12 for patients with doses < 0.5 Gy (Ron et al, 1992).
2. The Russian emergency workers of the Chernobyl accident who are included in the Russian National Medical Dosimetric Registry have annual medical check-ups. In the period 1986–1990, the age-adjusted thyroid cancer incidence in this cohort was larger by a factor of 2.6 than in the male Russian population (Ivannov et al, 1997).
3. A subcohort of the atomic bomb survivors (AHS) received biennial clinical examinations at RERF. The thyroid cancer incidence in the AHS subcohort was about twice that of the non-AHS participants (Thompson et al, 1994).

There are two reasons for a large effect as observed in the MRH study is not applicable to the present study. First, thyroid cancer among those who were exposed as children by the Chernobyl accident is predominantly of aggressive nature, which would become obvious independent of any screening. Second, a special recall and screening for a group of patients can be expected to be more effective than a mass thyroid surveillance, in which several hundred-thousand children are checked for many issues, one of which is the thyroid disease. Screening effects as observed in the other two studies are consistent with what was assumed in the present study.

### Risk estimates

In the birth cohort 1971–1985, 95% of the age-specific thyroid doses in the study population are in the range of 0.03–1.8 Gy. This range is larger by a factor of 6 as the typical 95% range of doses of inhabitants with the same age from a settlement (Goulko et al, 1998). Therefore, the linearity of the dose–response found in the analysis based on age-specific doses can be assumed to hold also for individual doses.

The results for the median values of the EARPD of the birth cohort 1971–1985 in the six sub-areas of the study are surprisingly consistent (Table 4). The results of the study of Jacob et al (1998) of 2.3 (95% CI 1.4–3.8) cases per $10^4$ person-year Gy for contaminated areas in Ukraine, Russia and Belarus are almost identical with the present study, although the study area and assumed background incidence are different. The confidence range of the EARPD in Jacob et al (1998) is smaller by a factor of 1.7 because dose estimates were assumed to be independent in each of the three countries.

The thyroid cancer risk after external exposures during childhood was assessed in a pooled analysis to 4.4 (95% CI 1.9–10.1) cases per $10^4$ person-year Gy (Ron et al, 1995). The best estimate of the present study is lower by a factor of 2. The difference is not statistically significant ($P$ = 0.097). Also, there is a reason for the difference of the point estimates: the excess thyroid cancer incidence after the Chernobyl accident was steeply rising in the observation period 1991–1995, the EARPD is expected to increase with longer observation times. The studies analysed by Ron and her colleagues (1995)

© 1999 Cancer Research Campaign had included observations extending to several decades after the exposure.

The median value of the ERRPD in the present study is higher by a factor of 3 than the best estimate of 7.7 (95% CI 2.1–28.7) obtained from observations over longer periods after external exposures (Ron et al, 1995). The difference is not statistically significant ($P$ = 0.075). A question remains as to how the ERRPD for thyroid cancer after the Chernobyl accident will change over longer observation times.

The difference of the EARPD for males in the present study to that for females is consistent with observations after external exposures in the age before 15, where a difference by a factor of 2.5 was found (Ron, personal communication).

The ratio of the ERRPD for males exposed during childhood to external exposures compared to that of females found in several studies was in the range of 0.2–2.6 (Ron et al, 1995). The ratio of 2 found in our study is additional information for this issue.

No plausible explanation could be found for the low number of observed cancer cases among males born in the period 1968–1975 as compared to females in the same birth cohort or to males born in the period 1976–1985. This phenomenon is not only observed in the study area but also in the whole of Belarus (Heidenreich et al, 1999). Military service does not seem to be an explanation because all cases occurring during this time have to be reported to the registry. Nevertheless, possible bias requires that results on age-at-exposure dependence should be treated with caution, as does the relatively low EARPD of children born in the period 1971–1975. This is possibly due to a more intense thyroid surveillance of school children.

In spite of these limitations, results of the present study on age-at-exposure dependencies of excess risks per unit thyroid dose are consistent with observations among the atomic bomb survivors: the EARPD for 0–10 years at exposure and 10–20 years at exposure was 4.37 resp. 2.67 cases per $10^4$ person-year Sv (Thompson et al, 1994).

Although the ERRPD has a large uncertainty, and the best estimates found in the present study for 5–9 years after an $^{131}$I exposure and in other studies for several decades after external exposures differ by a factor of 3, the age-at-exposure dependence in the different studies is similar (Figure 4).

### CONCLUSION

The excess absolute cancer risk per unit thyroid dose for the birth cohort 1971–1985 was found for the observation period of 4.6–9.6 years after the exposure to be 2.1 (95% CI 1.0–4.5) cases per $10^4$ person-year Gy. The point estimate is lower by a factor of 2 than what was observed in a pooled study of thyroid cancer risk after external exposures (Ron et al, 1995). Uncertainties of thyroid dose estimates have been identified as the main source of uncertainty of the EARPD. A main focus of future research should therefore be improving estimates of thyroid doses due to the Chernobyl accident.

The estimated ERRPD has a larger uncertainty than the EARPD, since the background incidence has a large uncertainty and contributes only a small portion to the observed cancer cases. In the context of the present study, EARPD and ERRPD are just numbers to describe the results, the observation period is too short to decide whether an absolute or a relative risk model describes better the thyroid cancer incidence after the Chernobyl accident.

The present study is an aggregate study using age-specific thyroid doses and thyroid cancer incidence among the population of three cities and 2729 settlements at the time of the Chernobyl accident. Indications are given that quantitative risk estimates could be obtained by the study: the dose–effect response is linear, the variability of individual doses in the single settlements is smaller than the variability of the average doses across the settlements, and the assumed effect range of intensified thyroid surveillance was shown not to influence strongly the estimates of the EARPD. Nevertheless, further methodological studies are needed to clarify the reliability of the risk estimates obtained.

Registry data about thyroid cancer cases have been used. Almost all Belorussian children with thyroid cancer were operated upon in the Republican Scientific and Pratical Center of Thyroid Tumors (Minsk) and pathological diagnosis was verified by international experts. However, the verification of the other case records used in this study is less clear. For a reliable assessment of the consequences of the Chernobyl accident, an inspection of the available samples from these other cases is highly desirable. The outcome of such inspections might well influence results of risk analyses.

In the present study, a relatively large effect of thyroid surveillance on the recorded thyroid cancer cases was assumed. The ERRPD is inversely proportional to the assumed effect of thyroid surveillance. The influence on the EARPD for the study area with high thyroid doses was shown to be small. However, thyroid cancer is also drastically enhanced in areas with lower thyroid doses. At least for studies in these areas, the question of the effect of thyroid surveillance has to be addressed again.

### ACKNOWLEDGEMENTS

This study was supported by the INCO-COPERNICUS project IC15CT960306 of the European Commission, and by the project ‘Scientists help Chernobyl children’ supported by the German Electricity Companies (VDEW). The authors are grateful to Dr TI Bogdanova, Prof. Dr IA Likhtarev and Prof. Dr ND Tronko for helpful discussions.

### REFERENCES

Astakova LN, Anspaugh LR, Beebe GW, Bouville A, Drozdovitch VV, Garber V, Gavrilin YI, Khrouch VT, Kuvshinnikov AV, Kuzmenkov YN, Minenko VP, Moschik KF, Nalivko AS, Robbins J, Shemiakina EV, Shinkarev S, Tochitskaya SI and Waclawiw MA (1998) Chernobyl-related thyroid cancer in children of Belarus: a case–control study. *Radiat Res* **150**: 349–356

Beral V and Reeves G (1992) Childhood thyroid cancer in Belarus. *Nature* **359**: 680–681

Buglova EE, Kenigsberg JE and Sergeeva NV (1996) Cancer risk estimation in Belarussian children due to thyroid irradiation as a consequence of the Chernobyl nuclear accident. *Health Phys* **71**: 45–49

Buglova EE, Demidchik E, Kenigsberg JE and Golovneva A (1997) Thyroid cancer in Belarus after the Chernobyl accident: incidence, prognosis of progress, risk assessment. In: *Low Doses of Ionizing Radiation: Biological Effects and Regulatory Control*, pp 280–284. IAEA: Vienna

Drozdovitch VV, Goulko GM, Minenko VF, Paretzke HG, Voigt G and Kenigsberg YI (1997) Thyroid dose reconstruction for the population of Belarus after the Chernobyl accident. *Radiat Environ Biophys* **36**: 17–23

Franssila KO and Harach HR (1986) Occult papillary carcinoma of the thyroid in children and young adults. *Cancer* **58**: 715–719

Gavrilin YI, Gordeev KI, Ivanov VK, Ilyin LA, Kondrusev AI, Margulis UY, Stepanenko VF, Khrouch VT and Shinkarev SM (1992) The process and results of the reconstruction of thyroid doses for the population of contaminated areas of the Republic of Belarus. *News Acad Med Sci USSR* **2**: 35–43

Goulko GM, Chepurny NI, Jacob P, Kairo IA, Likhtarev IA, Pröhl G and Sobolev BG (1998) Thyroid dose and thyroid cancer incidence after the Chernobyl accident: assessments for the Zhytomyr region (Ukraine). *Radiat Environ Biophys* **36**: 261–273

*© 1999 Cancer Research Campaign*

Greenland S and Robins J (1994) Invited commentary: ecologic studies – biases, misconceptions, and counterexamples. *Am J Epidemiol* **139**: 747–760

Hall P, Mattson A and Boice JD (1996) Thyroid cancer after diagnostic administration of iodine-131. *Radiat Res* **145**: 86–92

Hamilton P, Chiacchierini R and Kacmarek R (1989) *A Follow-up of Persons who had Iodine-131 and other Diagnostic Procedures during Childhood and Adolescence*. Publication FDA 89–8276. CDRH-Food and Drug Administration: Rockville, MD

Heidenreich WF, Kenigsberg J, Jacob P, Buglova EE, Goulko G, Paretzke HG and Demidchik EP (1999) Time trends of childhood thyroid cancer in Belarus. *Radiant Res* **151**: 617–625

Ivannov VK, Tsyb AF, Gorsky AI, Maksyutov MA, Rastopchin EM, Konogorov AP, Korelo AM, Biryukov AP and Matyash VA (1997) Leukemia and thyroid cancer in emergency workers of the Chernobyl accident; estimation of radiation risks (1986–1995). *Radiat Environ Biophys* **36**: 9–16

Jacob P, Goulko G, Heidenreich WF, Likhtarev I, Kairo I, Tronko ND, Bogdanova TI, Kenigsberg J, Buglova E, Drozdovitch V, Golovneva A, Demidchik EP, Balonov M, Zvonova I and Beral V (1998) Thyroid cancer risk to children calculated. *Nature* **392**: 31–32

James F (1994) *Minuit Function Minimization and Error Analysis*. CERN: Geneva

Kaidanovsky GN and Dolgirev EI (1997) Calibration of radiometers for mass control of incorporated $^{131}$I, $^{134}$Cs and $^{137}$Cs nuclides with the help of volunteers. *Radiat Prot Dosim* **71**: 187–194

Kazakov VS, Demidchik EP and Astakova LN (1992) Thyroid cancer after Chernobyl. *Nature* **359**: 21

Kerber RA, Till JE, Simon SL, Lyon JL, Thomas DC, Preston-Martin S, Rallison ML, Lloyd RD and Stevens W (1993) A cohort study of thyroid disease in relation to fallout from nuclear weapons testing. *JAMA* **270**: 2076–2082

Likhtarev IA, Sobolev BG, Kairo IA, Tronko ND, Bogdanova TI, Oleinic VA, Epshtein EV and Beral V (1995) Thyroid cancer in the Ukraine. *Nature* **375**: 365

McCullagh P and Nelder JA (1991) *Generalized Linear Models*. Chapman and Hall: London

Pacini F (1997) Post-Chernobyl thyroid carcinoma in Belarus children and adolescents: Comparison with naturally occuring thyroid carcinoma in Italy and France. *J Clin Endocrinol Metab* **82**: 3563–3569

Pierce AP, Stram DO and Vaeth M (1990) Allowing for random errors in radiation dose estimates for the atomic bomb survivor data. *Radiat Res* **123**: 275–284

Ron E, Lubin J and Schneider AB (1992) Thyroid cancer incidence. *Nature* **360**: 113

Ron E, Lubin JH, Shore RE, Mabuchi K, Modan B, Pottern LM, Schneider AB, Tucker MA and Boice JD (1995) Thyroid cancer after exposures to external radiation: a pooled analysis of seven studies. *Radiat Res* **141**: 259–277

Sheppard L, Prentice RL and Rossing MA (1996) Design considerations for estimation of exposure effects on disease risk, using aggregate data studies. *Stat Med* **15**: 1849–1858

Sobolev B, Heidenreich WF, Kairo I, Jacob P, Goulko G and Likhtarev I (1997) Thyroid cancer incidence in the Ukraine after the Chernobyl accident: comparison with spontaneous incidences. *Radiat Environ Biophys* **36**: 195–199

Stsjazhko VA, Tsyb AF, Tronko ND, Souchkevitch G and Baverstock KF (1995) Childhood thyroid cancer since accident at Chernobyl. *Br Med J* **310**: 801

Thomas D, Stram D and Dwyer J (1993) Exposure measurement error: influence on exposure–disease relationships and methods of correction. *Annu Rev Publ Health* **14**: 69–93

Thompson DE, Mabuchi K, Ron E, Soda M, Tokunaga M, Ochikubo S, Sugimoto S, Ikeda T, Terasaki M, Izumi S and Preston DL (1994) Cancer incidence in atomic bomb survivors. Part II: Solid tumors, 1958–1987. *Radiat Res* **137**: S17–S67

Ulanovsky AV, Minenko VF and Korneev SV (1997) Influence of measurement geometry on the estimate of $^{131}$I activity in the thyroid: Monte Carlo simulation of a detector and a phantom. *Health Phys* **71**: 34–41

Williams D (1994) Chernobyl, eight years on. *Nature* **371**: 556

Zvonova IA and Balonov MI (1993) Radioiodine dosimetry and prediction of consequences of thyroid exposure of the Russian population following the Chernobyl accident. In: *The Chernobyl Papers. Vol. I. Doses to the Soviet Population and the Early Health Effects Studies*, Mervin SE and Balonov MI (eds), pp. 71–125. WA Research Enterprises: Richland, WA

Zvonova IA, Balonov MI and Bratilova AA (1998) Thyroid dose reconstruction for population of Russia suffered after the Chernobyl accident. *Rad Prot Dosim* **79**: 175–178

*© 1999 Cancer Research Campaign*
